# Epileptic seizure induced by rapid repetitive limb movements

**DOI:** 10.1111/cns.13968

**Published:** 2022-09-07

**Authors:** Jianfang Zhang, Dengchang Wu, Caihong Ji, Yi Li, Xing Zhang, Liping Sun, Kang Wang

**Affiliations:** ^1^ Epilepsy Center, Department of Neurology, First Affiliated Hospital, School of Medicine Zhejiang University Hangzhou China

Dear Editor:

Reflex seizures (RSs) primarily refer to epileptic events that are triggered by external stimuli or an internal mental process.^1^ Reflex epilepsies (REs) are characterized by seizures that can be precipitated by numerous stimuli. Exercise‐induced seizure is a subtype of RS that is triggered by exercise. Most movement‐induced seizures are focal seizures without awareness, and the most common origin site of these seizures is the left temporal lobe.

Herein, we report the case of a patient with exercise‐induced seizures along with a review of previously reported cases with exercise‐induced RE (Table 1). Different from the seizures triggered by prolonged exercise reported in literature, the present patient's seizures generally appeared within 5–7 s of rapid repetitive limb movements. With trigger avoidance combined with antiseizure medications (ASMs), the patient was seizure‐free for 12 months.

**TABLE 1:** Features of previously published exercise‐induced cases

Authors, year	Gender, age	Seizure type	The latency from stimulus onset to evoked seizure	Spontaneous seizure	Neuroimaging lesion	Type of exercise	Medical treatment response
McLaren JR, 2021	Boy, 7	FAS	More than 30 s	Unknown	+	Cycling	unknown
Kamel JT, 2014	F, 28	FIAS	5 min	+	+	Cycling	Complete seizure control
Kamel JT, 2014	M, 49	FIAS, FBTCS	In the latter parts of his runs, after several kilometres	+	+	Running	Refractory
Kamel JT, 2014	M, 63	FIAS	Unknown	+	+	Running	Refractory
Kamel JT, 2014	F, 48	FIAS	Unknown	−	+	Playing netball	Refractory
Kamel JT, 2014	F, 50	FIAS	Unknown	+	+	Dancing	Refractory
Kamel JT, 2014	M, 74	FIAS	Unknown	+	+	Cycling	Refractory
Kamel JT, 2014	M, 44	FIAS, FBTCS	Unknown	+	+	Weight lifting	Refractory
Kamel JT, 2014	F, 27	FIAS, FBTCS	Unknown	+	+	Martial arts	Refractory
Kamel JT, 2014	M, 31	FIAS, FBTCS	Unknown	+	+	Cycling	Refractory
Kamel JT, 2014	M, 45	FIAS, FBTCS	At the end of his training	+	+	Running, cycling	Refractory
Werz, M.A, 2005	F, 21	GTCS	15 min	_	_	Exercise on a stair‐climbing machine	Complete seizure control
Sturm, JW, 2002	M, 16	FIAS	5–20 min	+	+	Running and playing soccer or tennis were more likely if the exercise was strenuous	Complete seizure control
Sturm, JW, 2002	M, 28	FIAS	5–10 min	+	+	Bicycle rides and races, more likely during strenuous exertion	Refractory
Schmitt B, 1994	Boy, 20 months	GTCS	Walking about 150 m	+	−	Walking	Refractory
Schmitt B, 1994	Boy, 4.5	GTCS	50 min	+	−	Playing football	Refractory
Simpson RK, 1989	Three adults	FIAS	Unknown	+	+	Jogging	Unknown
Ogunyemi AO, 1988	Boy, 14	GTCS	5–20 min	+	−	Running	Refractory
Ogunyemi AO, 1988	Boy, 7	FIAS	7 min	+	−	Riding a bicycle or swimming	Refractory
Ogunyemi AO, 1988	M, 21	GTCS	27 min	+	−	Strenuous exercise	Refractory

Abbreviations: −, negative test; +, positive test; FBTCS, focal to bilateral tonic–clonic seizures; FIAS, focal impaired awareness seizures; GTCS, generalized tonic‐–clonic seizure.

## 1 CASE REPORT

A 15‐year‐old right‐handed boy was referred to our epilepsy unit for diagnostic evaluation. The patient's first seizure occurred at the age of 9 years and commenced with numbness in the extremities soon after he starting running, which was followed by staring and oral and bimanual automatisms that lasted approximately 30 s before regaining consciousness. The patient remained asymptomatic without medication until the age of 12 years when he began experiencing the same symptoms while exercising. He noticed that physical activity was a reliable precipitation in seizure production. Interestingly, the seizures were triggered by rapid repetitive limb movements such as sprinting and fast pedalling and usually occurred within several seconds of initiating exercise. The seizure frequency increased after the age of 15 years, from two or three episodes per month to daily attacks. The patient experienced his first generalized tonic–clonic seizure in April 2021, which was independent of exercise, while he was awake.

**FIGURE 1:**
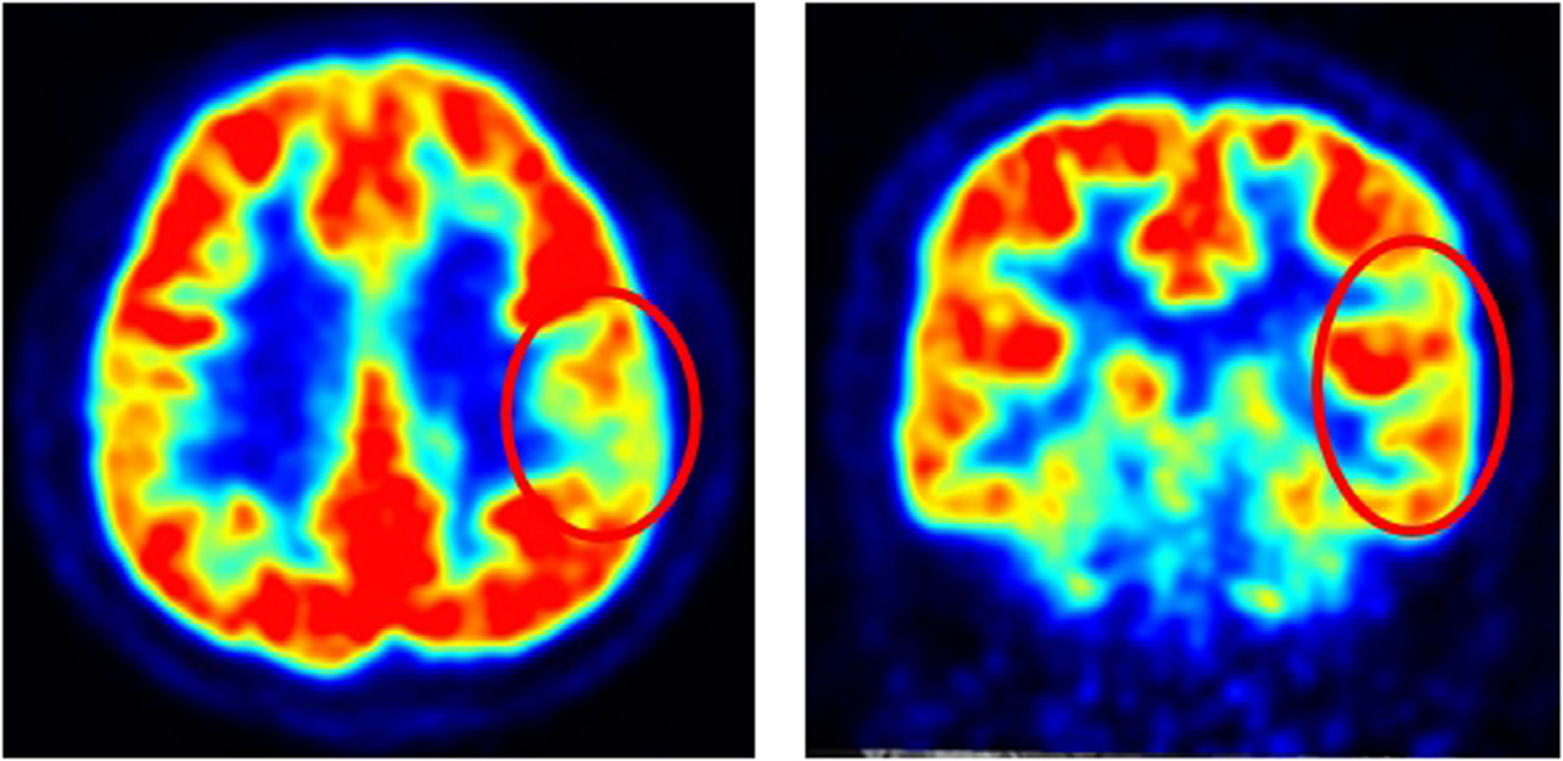
Axial and coronal slides of 18F‐fluorodeoxyglucose brain PET scan demonstrated hypometabolism regions (red circles) in left frontoparietal lobe.

**FIGURE 2:**
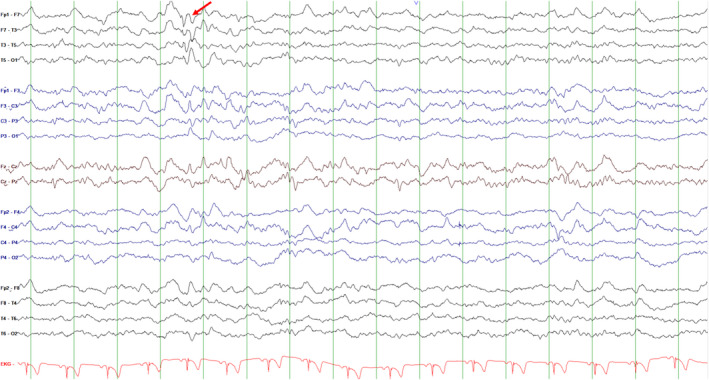
Interictal EEG demonstrating left temporal epileptiform discharges (red arrow).

The patient's birth was preterm but uneventful, with no family history of epilepsy. Neurologic and general physical examinations were normal but 18F‐fluorodeoxyglucose positron emission tomography (PET) scan revealed left frontoparietal lobe hypometabolism (Figure 1). During video‐EEG monitoring, interictal EEG detected abundant left fronto‐temporal epileptiform discharges (Figure 2). Seven exercised‐induced seizures (see the attached Video S1) and two spontaneous seizures were recorded. All RSs were elicited by rapidly swaying arms and simulating sprinting for 5 or 7 s. The frequency of arm swaying reached as high as 3.5–4 Hz. These recorded seizures clearly demonstrated the seizure onset from the left hemisphere, probably from the frontoparietal lobe. Approximately 80% of similar exercise patterns could trigger his seizure. He benefited greatly from the combination of topiramate and oxcarbazepine with exercise limitation. The patient was seizure‐free for 12 months, and a repeat EEG showed no interictal epileptiform discharges.

## 2 DISCUSSION

In this report, we presented the case of a boy with possible left frontoparietal lobe epilepsy induced by exercise as well as spontaneously occurred seizure. We discuss the possible roles through which exercise may precipitate seizures. The sine qua non of the seizure triggering is the existence of an epilepsy‐prone brain network to ignite a seizure that manifests the same system's functions.^2^ The cortical excitability may be increased in response to exercise, thereby reducing the seizure threshold and precipitating an event via certain cortical and subcortical pathways.

All events in our patient were focal seizures without awareness, which is consistent with previous data.^3^ In the present case, PET revealed hypometabolism in the frontoparietal lobe. Moreover, although the ictal EEG was not a classical manifestation of temporal lobe epilepsy, it revealed a clear ictal onset from the left hemisphere, with a likelihood origin from the frontoparietal lobe. However, existing literature suggests that exercise‐induced seizures typically originate from the temporal region, in which the left hemisphere is more commonly involved than the right.^4^, ^5^ The origin of such seizures in the frontoparietal lobe was rarely mentioned in literature. All patients experience provoked events, accompanied by spontaneous seizures, except one patient who had no seizure independent of exercise.

In the present case, the extent of physical exercise was proportional to that of the seizures, which were probably precipitated by vigorous rather than gentle exertion. Each patient had a unique exercise type that triggered the epileptic events. The previous instances of seizures in our patient were rather short, with triggers starting within 5–7 s after swaying arms rapidly. The pattern of such rapid movements apparently did not lead to fatigue, suggesting that high‐frequency limb movements raise cortical excitability and reduce the seizure threshold in certain manners. Therefore, seizures triggered by exercise were not only associated with the intensity of movement but also with the frequency of movement. High frequency was probably also a contributing factor to the seizures.

The management of RE is similar to that of other epilepsies. ASM are required for most of the patients. Unfortunately, despite the best management efforts, RS can be refractory in a substantial proportion of patients. Only a small fraction of these individuals who remain pharmoco‐resistant may benefit from surgical resection as postoperative outcomes are not generally favourable. As exercise‐induced seizures have more widespread epileptogenic network in terms of seizure initiation and propagation, surgically resecting the network is difficult. Other surgical options, including vagal nerve stimulation and deep brain stimulation, have been widely used in refractory epilepsy,^6^, ^7^ but their role in the treatment of exercise‐induced epilepsy has not been confirmed. These treatments might be considered as an option in the future. Additionally, microvascular changes, in which the vascular endothelial growth factor plays a key role, possibly occur in patients with epilepsy. Thus, evaluating the relationship between cerebral microvasculature and epilepsy may lead to the development of new therapeutic targets.^8^

## 3 CONCLUSION

Herein, we reported the case of an unusual RE triggered by rapid and repetitive limb movements, thereby expanding knowledge on the types of trigger patterns in exercise‐induced epilepsy. Considering the role of exercise in protection against seizure, physical exercise should be encouraged to most patients with epilepsy. However, this is not suitable for patients with exercise‐induced epilepsy, and limiting exercise or modifying the exercise habits may be beneficial for their seizure control.

## 4 AUTHOR CONTRIBUTIONS

Kang Wang has contributed to searching the literature and revising the manuscript. Jianfang Zhang is responsible for follow‐up of the patient, drafting the manuscript and clinical evaluation. Yi Li, Caihong Ji and Dengchang Wu have analysed the data and searched the literature. Liping Sun and Xing Zhang are in charge of following up the patient.

## 6 FUNDING INFORMATION

This work was supported by the Zhejiang Provincial Natural Science Foundation of China (LY19H090020).

## 7 CONFLICT OF INTEREST

The authors declare no conflict of interest.

8

## 9 CONSENT TO PARTICIPATE

Written informed consent was obtained from the parents.

## 10 CONSENT TO PUBLISH

The parents have consented to the submission of the case report to the journal.

## Supporting information


Video S1

## Data Availability

The data used during the current study are available from the corresponding author upon reasonable request.
